# Shape Fidelity Evaluation of Alginate-Based Hydrogels through Extrusion-Based Bioprinting

**DOI:** 10.3390/jfb13040225

**Published:** 2022-11-07

**Authors:** Mikail Temirel, Sajjad Rahmani Dabbagh, Savas Tasoglu

**Affiliations:** 1 Department of Biomedical Engineering, University of Connecticut, Storrs, CT 06269, USA; 2 Mechanical Engineering Department, School of Engineering, Abdullah Gul University, Kayseri 38080, Turkey; 3 Department of Mechanical Engineering, Koç University, Sariyer, Istanbul 34450, Turkey; 4 Koç University Arçelik Research Center for Creative Industries (KUAR), Koç University, Istanbul 34450, Turkey; 5 Koç University Translational Medicine Research Center (KUTTAM), Koç University, Istanbul 34450, Turkey; 6 Boğaziçi Institute of Biomedical Engineering, Boğaziçi University, Istanbul 34684, Turkey

**Keywords:** alginate, bioink, bioprinter, extrusion, gelatin, shape fidelity

## Abstract

Extrusion-based 3D bioprinting is a promising technique for fabricating multi-layered, complex biostructures, as it enables multi-material dispersion of bioinks with a straightforward procedure (particularly for users with limited additive manufacturing skills). Nonetheless, this method faces challenges in retaining the shape fidelity of the 3D-bioprinted structure, i.e., the collapse of filament (bioink) due to gravity and/or spreading of the bioink owing to the low viscosity, ultimately complicating the fabrication of multi-layered designs that can maintain the desired pore structure. While low viscosity is required to ensure a continuous flow of material (without clogging), a bioink should be viscous enough to retain its shape post-printing, highlighting the importance of bioink properties optimization. Here, two quantitative analyses are performed to evaluate shape fidelity. First, the filament collapse deformation is evaluated by printing different concentrations of alginate and its crosslinker (calcium chloride) by a co-axial nozzle over a platform to observe the overhanging deformation over time at two different ambient temperatures. In addition, a mathematical model is developed to estimate Young’s modulus and filament collapse over time. Second, the printability of alginate is improved by optimizing gelatin concentrations and analyzing the pore size area. In addition, the biocompatibility of proposed bioinks is evaluated with a cell viability test. The proposed bioink (3% *w*/*v* gelatin in 4% alginate) yielded a 98% normalized pore number (high shape fidelity) while maintaining >90% cell viability five days after being bioprinted. Integration of quantitative analysis/simulations and 3D printing facilitate the determination of the optimum composition and concentration of different elements of a bioink to prevent filament collapse or bioink spreading (post-printing), ultimately resulting in high shape fidelity (i.e., retaining the shape) and printing quality.

## 1. Introduction

The three-dimensional (3D) bioprinting technology refers to the layer-by-layer patterning of cell-laden bioink(s) in a predefined structural design [1,2,3]. Applications of 3D bioprinting range from microfluidics, organ-on-chip technologies, and tissue engineering to real-sized organ implants [4,5,6,7,8,9,10,11,12,13,14,15,16,17,18,19,20,21]. Commonly used 3D bioprinting methods are laser-assisted bioprinting [22], stereolithography (SLA) [17,23], inkjet-based bioprinting [24], valve-based bioprinting [25], and extrusion-based bioprinting (EBB) [26]. While laser-assisted and SLA (known as light-induced methods) possess high resolution and can fabricate complex 3D patterns, the high cost, limited material selection, scalability, and potential photo-induced cell damages are challenging. On the other hand, although inkjet-, valve-, and extrusion-based methods (known as nozzle-based methods) are more cost-effective and readily available, mediocre resolution and shear stress during the printing process (resulting in lower cell viability) are major predicaments to be addressed [27].

The EBB is one of the most prevalently used 3D bioprinting methods, ranging from organ-sized models to organ-on-chip platforms [16,28,29] to cell-laden scaffolds [30,31], due to the ease of operation, acceptable resolution, and multi-material printing ability (multi-nozzle and/or co-axial printing [26,30,31,32]). However, the main challenges faced by EEB are: large shear stress on the cell during deposition (decreasing cell viability [33,34]), deformation (limiting the ability to produce complex 3D patterns with overhanging structures), and bioink dispersion post-printing (reducing the printing resolution). While bioinks with higher viscosity can partially address the dispersion and deformation issues, nozzle clogging and nonuniform bioink flow are the conceivable repercussions of more viscose bioinks, indicating the importance of bioink properties and composition in successful bioprinting.

An important step in developing a new bioink is to assess shape fidelity and printability. Printability refers to the ability of a material to be deposited in a predesigned pattern (i.e., similar to CAD design) while retaining its initial shape (i.e., shape fidelity) [33,35,36]. The assessment of the shape fidelity of a single filament is a major task to optimize the functionality of bioprinting and is required to estimate printing accuracy. Bioinks used in EBB should possess shear-thinning behavior (i.e., a decrease of viscosity with an increase in shear rate [37]). Moreover, cell viability is another important point to be considered, as cells can be damaged during the printing process (by shear or thermal stresses) or post-printing (by filaments with low biocompatibility). Although high-viscosity bioinks can maintain the 3D-bioprinted pattern during the incubation period, these bioinks experience a higher shear force during printing, decreasing cell viability [38,39]. Natural hydrogels, including alginate [40], gelatin [41,42,43], collagen [44], and chitosan [45], have been commonly used for bioprinting due to their similarities to native extracellular matrix (ECM). However, the mechanical properties of these natural bioink components are generally poor in terms of printability. This limitation can be mitigated by adjusting the materials’ properties through additive materials [46,47].

There were attempts to determine the printability and shape fidelity of bioinks quantitatively. In this regard, the degree of pore circularity in a grid pattern is used as a measure of high printability (i.e., less circularity post-printing means more similarity of print outcome to the computer design) [38]. Nonetheless, this method focuses only on the X–Y resolution of patterns, overlooking the deformation of hanging filaments and bioink rheology in more complex designs. In order to determine whether a filament can retain its shape after being printed, different compositions and concentrations of bioinks were printed on support structures with varying distances between them [48,49,50,51]. Although early studies enabled experimental determination of the proper composition of a bioink to be able to avoid filament collapse in the desired bridging distances, these studies lacked quantitative analysis which can correlate the bioink properties (e.g., viscosity) to the shape fidelity of the bioink. The deformation angle of hanging bioinks was studied quantitatively using 3D printing of suspending filaments with different combinations of poloxamer 407 with poly(ethylene glycol) (PEG) [35]. The developed model overestimated the deflection angle, yet the slope of the fitted lines for the theoretical values resembled the same trend of experimental results. This model presented a reproducible method for testing new material formulations and comparing them with established ones [35]. While examination of the structural properties of bioinks is crucial for the fabrication of more complex patterns, the biological performance of the bioinks (e.g., cell viability) and their reaction to real-life scenarios (e.g., at body temperature) should also be assessed.

Herein, two quantitative assessments of the printability and shape fidelity of alginate-based bioink are performed. First, the filament collapse is evaluated to estimate the filament deformation in the case of printing by different temperatures, alginate concentrations, and over supports with varying spacing. Additionally, a theoretical model is developed to predict Young’s modulus of the filament as a function of its radius. Finite element analysis was performed to validate the theoretical model. Moreover, the poor printability of alginate is improved by adding different concentrations of gelatin. In this regard, two-layer and six-layer grid patterns are printed with alginate–gelatin combinations, and the pore area between the grid patterns is measured in three phases (after printing, crosslinking, and at two days of incubation) in order to determine the optimum combination that results in the best shape fidelity and possible effects of the incubation on shape fidelity. Finally, the biocompatibility of the proposed bioinks is evaluated with cell viability tests. All 3D-printed filaments were incubated at 37 °C (i.e., resembling body temperature) to characterize the effects of the temperature on the filament diameter, deformation, swelling, and/or contraction over time. This can pave the path for a better understanding of the fidelity of bioprinted structures under actual in vivo conditions for the development of in situ 3D bioprinting of wound healing patches in the near future. Moreover, the proposed mathematical model correlates filament deformation and radius with Young’s modulus.

## 2. Material and Methods

### 2.1. Hydrogel Preparation

Alginate bioink was prepared as described in the literature [52]. In summary, sodium alginate powder (Sigma-Aldrich, St. Louis, MO, USA) was dissolved in phosphate-buffered saline (PBS 1X, pH 7.4, Sigma-Aldrich, St. Louis, MO, USA) at concentrations of 2%, 4%, 6%, and 8% (*w*/*v*). Alginate–gelatin (from bovine skin, type B, Sigma-Aldrich, St. Louis, MO U.S) (Alg-Gel) bioinks were prepared by dissolving varying concentrations of gelatin powder (1%, 2%, and 3% *w*/*v*) with 4% *w*/*v* alginate powder in PBS. A 4% alginate concentration was selected for the mixture due to its cell viability, as reported in the literature [52]. The bioinks were vortexed (Cole-Parmer, Vernon Hills, CT, USA) for one minute at 3400 rpm, then restored at 37 °C for one day to fully dissolve the powder(s). For better visualizations post-printing, red food dye was added to the bioink mixture and vortexed for 30 s to increase the contrast, then held for 30–60 min at 37 °C to release any air bubbles before loading it into the syringe barrel. Bioinks were crosslinked in calcium chloride (CaCl_2_) (Sigma-Aldrich, St. Louis, MO, USA). This ionic crosslinker was dissolved in PBS at a concentration of 2% (*w*/*v*) by vertexing for one minute, followed by loading it into a syringe barrel.

### 2.2. Setup and Printing Parameters

**Figure 1:**
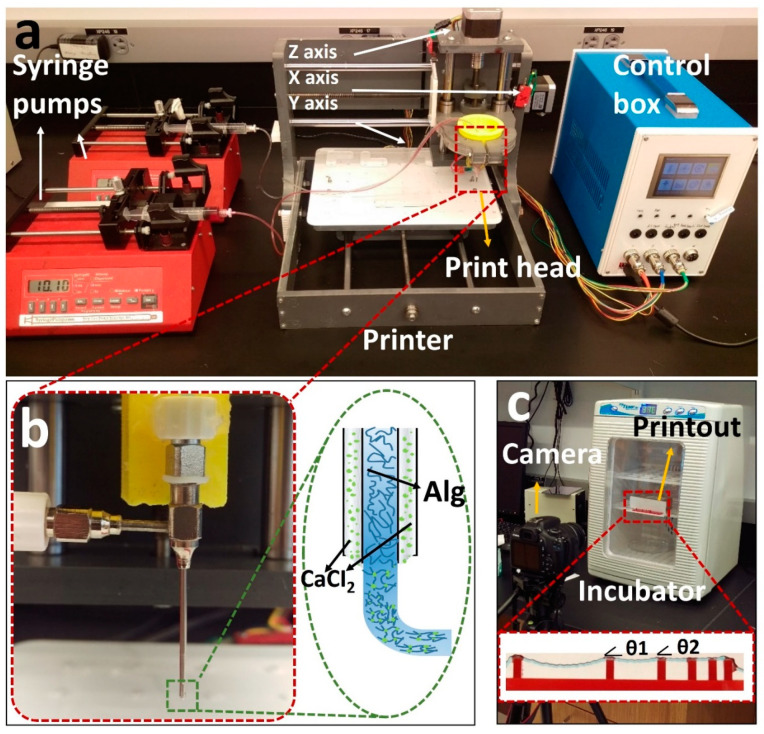
Overview of our custom-designed experimental setup. (**a**) Left to right: two commercial syringe pumps to extrude the hydrogel and crosslinker, a custom-made bioprinter using a CNC stage as a platform and a custom-made controller unit controlled via P.C. (**b**) Co-axial needle and schematic illustration of the printing process. Cell–alginate mixture and crosslinker (CaCl_2_) were deposited from the inner and outer needles, respectively. (**c**) Mini digital incubator with a camera to image the filament deformation.

A previously developed [52] custom-designed 3D bioprinter is used to perform the experiments (Figure 1a) [52]. Briefly, the bioprinter was made from a gantry-type three-axis CNC stage controlled by open-source Marlin firmware. A custom-designed head holder is used to hold a co-axial syringe tip (Rame-hart Instrument Co., Succasunna, NJ, USA), with a 22G inner tip and 18G outer tip. To deposit the bioink and crosslinker, two NE-4000 syringe pumps (New Era Pump Systems, Farmingdale, NY, USA) were connected to the co-axial syringe tip with silicone tubing. The syringe barrels were prepared (as described in the previous section) with alginate (inside inner tip) and a 2% *w*/*v* calcium chloride solution (inside outer tip) with flow rates of 0.04 and 0.01 mL/m, respectively, with a nozzle feed rate of 4 mm/s. The system was designed so that the calcium solution surrounded the alginate during extrusion to initiate the crosslinking (Figure 1b).

For the pore area analysis section, pneumatic extrusion (KLT-982A Auto Dispenser, Taiwan) was chosen, as it has less noise at lower flow rates compared with a syringe pump. Syringe pumps also suffer from pressure buildup, in which the bioink flow continues for a short time after the pump stops, causing additional deformity in the pattern. A 30 G nozzle tip, instead of the co-axial nozzle, was chosen to achieve a more precise pattern. The optimum nozzle speed and air pressure were experimentally determined (based on the concentrations) and were set to be 6 mm/s and 15 psi throughout the experiments, respectively.

The resolution of the utilized custom-designed 3D bioprinter was previously determined [52] by image analysis of the actual position of droplets compared with their intended position. Using an imaging setup with the resolution of 45 pixels per millimeter, the standard deviation of the bioprinter was measured to be within the range of 1–2 pixels, which means a ±44 μm resolution in the X–Y direction. In addition, to minimize the error of experiments, all printings were repeated three times, and their standard deviations are shown in the figures as error bars.

### 2.3. Filament Deformation Test

The filament deformation test is based on previous works to assess the mid-point deflection of a hanged filament [11,48]. A custom platform of 1.6 × 2.75 × 4 mm (length, width, and height) with pillars placed at 1, 2, 4, 8, and 16 mm along the platform was designed in SolidWorks (Dassault Systèmes SolidWorks Corp., Waltham, MA, USA). The design was cut with a commercially available laser cutter (VLS2.30 CO_2_ laser cutter; Universal Laser Systems, Inc., Scottsdale, AZ, USA) from an acrylic sheet (McMaster-Carr, Princeton, NJ, USA). Filaments made of different concentrations of alginate (2%, 4%, 6%, and 8% *w*/*v*) were deposited on the platform with the co-axial tip while the process was recorded (via a Canon DSLR camera) until the filament straightened horizontally due to evaporation. G-code for 2D bioprinting was generated manually using Repetier-Host (version 1.6.2, Hot-World GmbH & Co. K.G., Willich, Germany), a free-license 3D printer control software. To determine the effect of temperature on deformation, the experiment was repeated in an incubator (H220-H, Benchmark Scientific, San Diego, CA, USA) at a temperature of 37 °C. The platform was placed in the incubator immediately after printing. Images were then taken through the incubator’s transparent lid, focused on the middle point of the platform as seen in Figure 1c. Deformation angles θ1 and θ2 and filament diameter (Figure 1c) were measured via ImageJ. Three replicates were performed for each alginate concentration at both room temperature (25 °C) and 37 °C.

### 2.4. A Mathematical Model for Filament Deformation

A model is proposed to correlate filament deformation with Young’s modulus and filament radius. This model can be used to predict filament deformation. The following assumptions were made: (i) no deformation from gravity force within 20 s after printing, (ii) the cross-section area of filament remained constant, (iii) filament density remained constant, and (iv) no outside vibrations. The “Euler–Bernoulli simply supported beam” equation was used to describe the shape of the filament. The general governing differential equation is as follows:

(1) $$\frac{\partial^{4}w}{\partial x^{4}}=\frac{P}{EI}\frac{\partial^{2}w}{\partial x^{2}}+\frac{\rho A}{EI}\frac{\partial^{2}w}{\partial t^{2}}=\frac{q\left(x\right)}{EI}\;$$

where w(x,t) is the displacement of filament from the neutral axis as a function of position along the filament (*x*) and time (*t*). P is “transfer loading” caused by an adjacent beam. E is Young’s modulus, I is the moment of inertia for cylinder I=πr44, ρ is the density of bioink (~1026 kg/m^3^ for 4% alginate), A is the cross-section area, and q(x) is transverse distributed load (i.e., the force due to gravity).

Separating the displacement function $w\left(x,t\right)=e^{i\omega t}$, Equation (1) becomes:

(2) $$\left(\frac{\partial^{4}w}{\partial x^{4}}-\frac{P}{EI}\frac{\partial^{2}w}{\partial x^{2}}+\frac{\rho A\omega }{EI}w\right)e^{i\omega t}=\frac{q\left(x\right)}{EI}\;$$

where ω is the oscillation coefficient. No oscillation is assumed in the model, so ω=0. Equation (2) then reduces to:

(3) $$\frac{\partial^{4}w}{\partial x^{4}}-\frac{P}{EI}\frac{\partial^{2}w}{\partial x^{2}}=\frac{q\left(x\right)}{EI}\;$$

Equation (3) is divided into two solutions:

(4) $$\frac{\partial^{4}w}{\partial x^{4}}-\frac{P}{EI}\frac{\partial^{2}w}{\partial x^{2}}=0\;$$

(5) $$\frac{\partial^{4}w}{\partial x^{4}}-\frac{P}{EI}\frac{\partial^{2}w}{\partial x^{2}}=\frac{q\left(x\right)}{EI}\;$$

where (4) is homogenous, and (5) is particular. For the homogenous solution, λ and w are set to be: ∂w∂x=λ and $w=e^{\lambda x}$. Thus, Equation (4) becomes:

(6) $$w\left(x\right)=c_{1}+c_{2}x+c_{3}e^{\sqrt{\frac{Px}{EI}}}+c_{4}e^{-\sqrt{\frac{Px}{EI}}}\;$$

For Equation (5), the general solution for a fourth-order ordinary differential equation is $w=ax^{2}$. Solving this equation gives:

(7) $$w\left(x\right)=-\frac{q}{2P}x^{2}\;$$

Combining the homogenous solution (Equation (6)) and particular solution (Equation (7)), the final displacement equation becomes:

(8) $$w\left(x\right)=c_{1}+c_{2}x+c_{3}e^{\sqrt{\frac{Px}{EI}}}+c_{4}e^{-\sqrt{\frac{Px}{EI}}}-\frac{q}{2P}x^{2}\;$$

This gives us maximum deflection at x=L/2, the middle point of the filament. Using boundary conditions w(0)=w(L)=0 and $w^{\prime \prime }\left(0\right)=w^{\prime \prime }\left(L\right)=0$ for simply-supported beam, coefficients were found for each time period.

Along with an analytical and experimental examination of hanging filaments, the use of computational simulation via FEM can facilitate time-dependent modeling and visualization of hanging structures more accurately, in a shorter time, and with lower cost [53,54].

### 2.5. Bioink Rheology

Rheological characterization experiments of alginate–gelatin bioinks were performed on a rotational AR-G2 rheometer (T.A. Instrument, USA) using a 2° cone plate with a diameter of 40 mm and a gap of 150 µm. Plate temperature was kept constant at 25 °C (room temperature). The shear rate varied from 0.01 to 100 s^−1^. The linear viscoelastic region (LVR) was determined using 0.1% strain with a dynamic strain sweep. Oscillatory measurements of storage (G’) and loss (G”) modulus were conducted as a function of angular frequency ranging from 0 to 100 rad/s.

Compression testing was performed using a dynamic mechanical analyzer (DMA, TA Q800 TA Instrument, New Castle, DE, USA). The ramp force was set to 1 N/mm at 18 N and 25 °C. Prepared samples (10 × 10 mm and 2 mm in height) were placed on a uniaxial parallel plate with a diameter of 15 mm. The compressive modulus was calculated from the linear region of the stress–strain curve. Three replicates were performed for each time point.

### 2.6. Characterization of Pore Area

In order to determine the optimum concentration to achieve the best resolution in grid pattern bioprinting, alginate concentration was set to 4% *w*/*v* to be mixed with gelatin to improve the alginate’s printability. Gelatin concentrations of 1, 2, and 3% *w*/*v* were examined to determine the effects of viscosity on structural fidelity. A concentration of 3% *w*/*v* gelatin was set to be the upper limit for chosen alginate concentration, as a higher viscosity was not printable using the aforementioned tip size and air pressure. Pore size analysis was used to measure the successfulness of the bioprinting process (similar to CAD) and the area within the printed grid pattern to determine the effects of crosslinking and incubation on the pore size in a multi-layered design. A square grid pattern (20 × 20 mm with 11 × 11 = 121 pores) was printed in a glass slide (30 × 30 mm). Then, it was submerged in a 2% *w*/*v* calcium bath for two minutes for crosslinking. The crosslinked patterns were submerged in cell culture media at 37 °C for two days. Subsequently, the patterns were stained with red dye for better imaging. The images were then analyzed via ImageJ to measure the pore area.

### 2.7. Cell Printing Procedure

Bioinks were prepared in sterile conditions, with 2 × 10^6^ 3T3 NIH mouse fibroblast cells, and mixed with the bioinks immediately prior to bioprinting (the bioink preparation procedure is described in previous sections). The bioink–cell mixture was deposited in a one-layer pattern. For cell viability tests of grid patterns, the bioink was extruded on glass slides in a grid pattern. Following the print, the samples were transferred into a well plate on the same glass slide in order to avoid breakage or deformation of the printouts. Patterns were then crosslinked for 2 min using a 2% *w*/*v* CaCl_2_ solution. After removing the calcium solution, warm PBS (at 37 °C) was applied immediately, followed by two washes with cell media, and the samples were finally incubated in cell media. Cell viability experiments were performed with a gelatin–alginate mixture using a 30 G nozzle (not a co-axial nozzle). Fabrication of a multi-layered 3D grid pattern is challenging with a co-axial tip because once CaCl_2_ and alginate contact each other, alginate crosslinks (solidified) and loses its stickiness properties—meaning that the new alginate layer does not stick to the bottom layer to make a multilayer scaffold (bottom layer is already solidified). Alternatively, the extrusion of bioink with a single needle tip enables the stacking of multilayer 3D constructs with stuck layers.

### 2.8. Viability Characterization

Calcein AM (live green stain) and ethidium homodimer-1 (EthD, dead red stain-Life Technologies) were used to determine the cell viability. Stained cells were observed under a fluorescence microscope (Zeiss AXIO). The samples were firstly washed with PBS, followed by applying a solution of 1:2000 calcein and 1:500 ethidium homodimer in PBS. Each sample was submerged in the staining solution and incubated for 15 min. Images were taken over a z-axis range of 100 μm with six different focal planes spaced evenly across the z-range. Cell viability was quantified by combining the six images in a z-stack. The maximum value of each (x, y) pixel across all six planes was calculated and used to create a z-projection image for each channel. The “find maxima” function in ImageJ was used for each separate channel and each z position within the stack. Noise tolerance was set to 20. Each local maximum in the green channels (calcein) was considered a live cell, and those in the red channels (EthD) were taken as dead cells. Viability in each image was calculated as:

(9) $$\frac{\left(live\;cells\right)}{\left(live+dead\;cells\right)}\times 100\;$$

The mean viability for each crosslinking time was taken as the total number of live cells divided by the total number of cells (live and dead) counted across several images from two different bioprints. The composite images shown are pseudo-colored to show both calcein and EthD staining in a single image.

### 2.9. Statistical Analysis

Statistical analysis was performed to evaluate the significance of differences in the obtained results with “ANOVA single-factor” tests for multiple comparisons and “*t*-test: paired two-sample for means” for two-sample comparisons. The “ANOVA” method calculates the statistical differences by testing for differences of means using variances, while “the *t*-test” determines whether the mean difference between two sets of observations is zero. The analysis was conducted with Microsoft Excel 365 (2021). A value of *p* < 0.05 was considered to state statistical significance. All quantitative data were presented as mean ± standard deviations.

## 3. Results and Discussion

### 3.1. Theoretical Model and Finite Element Modeling of Filament Deformation

**Figure 2:**
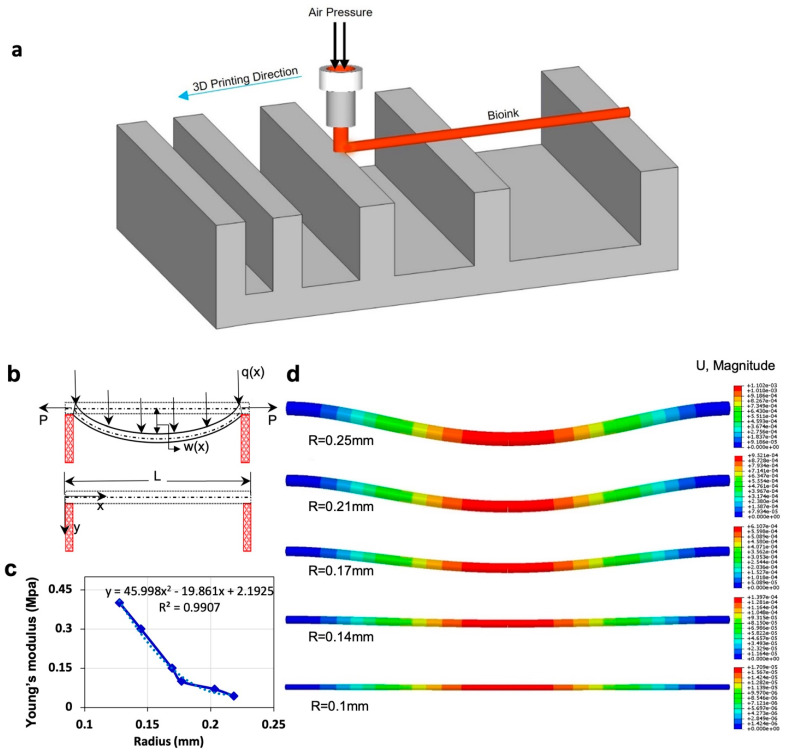
Mathematical modeling and finite element analysis (FEA). (**a**) Schematic view of filament deformation test. The filament is bioprinted on a support structure with varying distances. (**b**) Schematic diagram of filament deformation experiment. When the filament is deposited over the two supporting pillars, maximum deformation is observed at the midpoint due to gravity. W(x) is the maximum displacement of the filament in the middle of the filament. P is “transfer loading” of the filament that is caused by adjacent filament, and q(x) is transverse distributed load, which is a gravity force in our model. (**c**) Results of the theoretical model by using the experimental data of incubation condition. This is a correlation between Young’s modulus and the radius of the filament. (**d**) Results of FEA show the displacement of the filament due to gravity over time, at which the diameter of the filament decreased because of evaporation. Data from the mathematical model were used in the modeling. FEA was performed in Abaqus.

Figure 2c represents the relation between Young’s modulus and radius based on the theoretical model for 4% alginate at 37 °C. Young’s modulus of material increases as the filament radius decreases. As evaporation occurs from the filament, the ratio of solid to liquid increases, increasing Young’s modulus value. The dotted line represents the linear regression, giving the correlation between Young’s modulus and filament radius (R^2^ = 0.99), which assesses the elasticity modulus of filament in the condition of the known radius. Finite element analysis (FEA) modeling was performed using displacement magnitude for varying radii (Figure 2d). FEA replicated the experimental result by using data from the model. Upon application of the experimental data in the FEA, the displacements are obtained, which are shown in the legend bar next to each of the images and are consistent with the experimental results.

### 3.2. Filament Deformation Test

**Figure 3:**
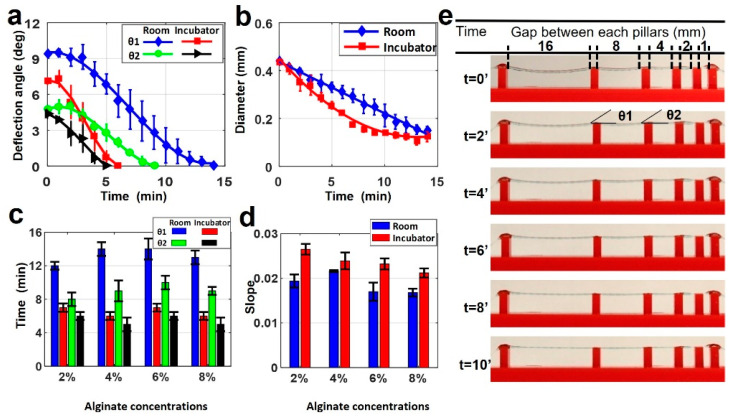
Characterization of filament deformation in room and incubator temperatures. (**a**) A comparison of two deflection angles on the filament in room and incubator temperatures (37 °C) for 4% alginate (*w*/*v*) concentration. (**b**) A comparison of filament diameter changing in room and incubator temperature over time for 4% alginate (*w*/*v*) concentration. *p*-value is 0.00098 (ANOVA: single-factor). (**c**) Total time in which the deflection angles reach zero for both θ1 and θ2 deflection angles in room and incubator temperature for four different alginate concentrations. *p*-value is 8.89 × 10^−6^ (*t*-test: paired two-sample for means). (**d**) Positive values of slopes correlating the diameter versus timeline as seen in b for both room and incubator temperature for four different alginate concentrations. *p*-value is 0.973 (ANOVA: single-factor). (**e**) Representative images of printed filament over the platform at incubator temperature for 4% alginate. Deflection and diameter changes are displayed over ten minutes. *p*-value is 0.679 (ANOVA: single-factor).

Filament deformation testing was conducted using the alginate-only bioinks. Samples were prepared as described above with varying pillar distances on the bioprinting surface. Once deposited and crosslinked, samples were kept at two different temperatures for imaging in order to determine the possible effects of temperature on deformation. Figure 3a depicts a comparison of two deflection angles for 4% alginate filament at 25 and 37 °C. No deflection was observed in gap lengths below 4 mm. At 37 °C, only 6 min was needed for the θ1 angle to drop to 0°, while it took 14 min at 25 °C. Similarly, the time for θ2 to drop to 0° was approximately 9 min at 25 °C, while it took 5 min at 37 °C. This was because of the increased evaporation rate due to the higher temperature and lower relative humidity. As the filament diameter is directly related to the water content (Figure 3b), temperature de facto affects the diameter of the filament as well. It is worth noting that the diameter further decreased even after the deflection angles reached zero. The deformation of the filament caused by gravity reached the equilibrium point with force against the deformation caused by Young’s modulus of filament.

Figure 3c demonstrates the total time in which the deflection angles (θ1 and θ2) reached zero in room and incubator temperature for the four different alginate concentrations. Alginate concentration slightly affected the total time to reach zero angles, approximately 20% at room temperature and 12% at incubator temperature for both angles. Figure 3d shows the positive values of slopes correlating the diameter versus timeline, as seen in Figure 3b for both room and incubator temperature for the all-alginate concentrations. These results also showed that temperature effects on the diameter occur for different alginate concentrations. The change of diameter in the incubator had a bigger slope than at room temperature for all concentrations. This indicates that the diameter in the incubator decreased faster than the diameter at room temperature due to fast evaporation, as explained above. Representative images of printed filament with 4% alginate in the incubator are shown in Figure 3e. This shows the deflection and diameter changing over ten minutes.

### 3.3. Rheological Characterization

**Figure 4:**
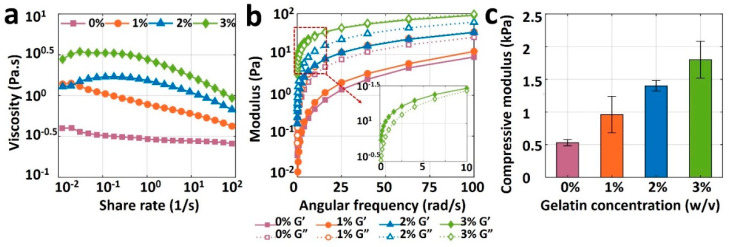
Results of rheological and mechanical characterization of bioinks. (**a**) Viscosities of 4% pure alginate (0% gelatin), and alginate with 1%, 2%, and 3% (*w*/*v*) gelatin, as a function of shear rate ranging from 0.01 to 100 s^−1^ (**b**) Storage modulus (G’) and loss modulus (G”) of all bioinks as a function of angular frequency ranging from 0 to 100 rad/s. (**c**) Compressive modulus of 3D-bioprinted and crosslinked samples with four different bioinks. A square of 10 mm-wide and 2 mm-thick sample was placed on the platform of dynamic mechanical analysis (DMA), and compression was performed until samples were yielded. The error bar is the standard deviation of 3 independent tests. *p*-value is 0.0015, ANOVA: single-factor.

Figure 4a depicts the viscosity measurement results for pure alginate (0% gelatin) and its mixture with three different gelatin concentrations as a function of share rate. The viscosity curve for pure alginate (viscosity against shear rate) is relatively flat, indicating the unsuitability of pure alginate for bioprinting, as the bioink needs to have a shear-thinning property. Although adding 1% or 2% *w*/*v* gelatin increased the viscosity of the bioink, it was not enough to retain the shape post-extrusion. Adding 3% gelatin, however, increased the viscosity, improving the printability and ability of the bioink to preserve its shape after deposition. The results of storage modulus, G’, and loss modulus, G”, of bioinks are depicted in Figure 4b. The loss moduli of 0, 1, and 2% gelatin were higher than their storage modulus across the entire angular frequency range of 0–100 rad/s, which indicates the liquid-like behavior that cannot protect shape after printing (low shape fidelity). Nonetheless, 3% gelatin had a higher storage modulus than its loss modulus, resulting in a solid-like material which can retain its shape when it is deposited. Results of the compression test are shown in Figure 4c for all bioinks. Compressive modulus increased linearly, from 0.5 to 1.8 kPa, with an increase in the gelatin concentration from 0 and 3% gelatin, respectively. As a result, the rheological and mechanical features of bioink made with pure alginate were insufficient for bioprinting due to its low viscosity and compressive modulus. Those properties were easily improved to achieve high shape fidelity with the addition of gelatin. A concentration of 3% gelatin in 4% alginate showed ideal rheology and mechanical strength for potential bioprinting purposes.

### 3.4. Pore Area Analysis

**Figure 5:**
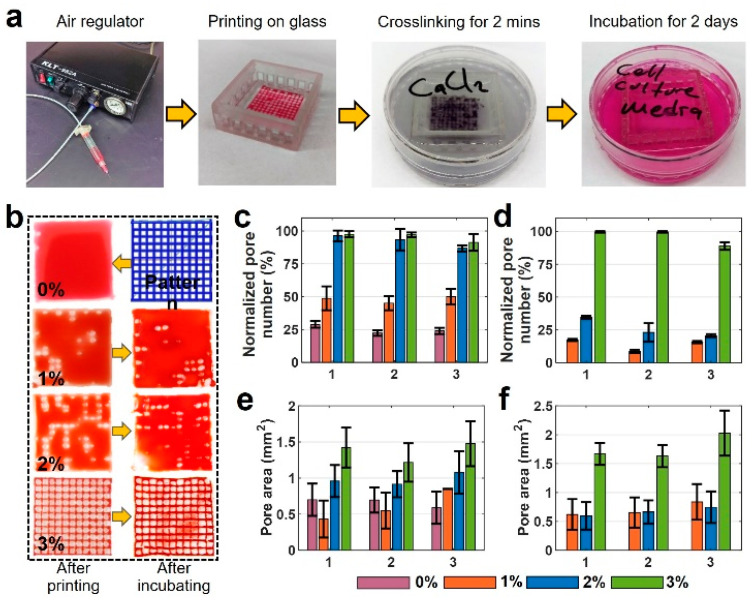
Characterization of print quality and pore area for multiple-layered grid pattern. (**a**) Sequential steps of the experiment. (**b**) Images of a 6-layer printed pattern with four different bioink mixtures. The first column shows the images that were taken after printing, and the second column shows those after two days of incubation. (**c**,**d**) Normalized pore number that shows the percentage of the successfully printed number of the pores after printing, after crosslinking, and after two days of incubation: (**c**) for 2-layer print and (**d**) for 6-layer print. (**e**,**f**) Pore area measurements: (**e**) 2-layer print and (**f**) 6-layer print after printing, after crosslinking, and after two days of incubation. There are no results of 0% for six layers as the bioink filled the whole pores in a grid pattern as seen in a. (x-axis for plots are 1: after printing, 2: after crosslinking, and 3: after incubating). For Figure 5c, *p*-value is 0.807 (ANOVA: single-factor); for Figure 5d, *p*-value is 0.968 (ANOVA: single-factor); for Figure 5e, *p*-value is 0.825 (ANOVA: single-factor); for Figure 5f, *p*-value is 0.876 (ANOVA: single-factor).

Representative images and quantitative results of pore area measurement analysis are depicted in Figure 5. Figure 5a depicts the sequential steps of the experiment. Figure 5b illustrates images of the six-layer printed pattern with four different bioink mixtures. The first column shows the images that were taken after printing, and the images in the second column are those taken after two days of incubation. The remainder of the experimental images for all concentrations and layers are available in Figure S2. According to Figure 5b, the 4% pure alginate could not keep its shape and spread around easily due to its low viscosity, filling in the pore area after deposition. The print quality was improved by adding gelatin (e.g., 1, 2, and 3% *w*/*v*), compared with pure 4% *w*/*v* alginate. For gelatin concentrations above 3% *w*/*v*, the high viscosity of the bioink precluded the bioprinting process. The quantification of experimental images is presented in Figure 5c–f. Figure 5c,d show normalized pore number—the percentage of successfully printed pores of two-layer print and six-layer print, respectively, after printing (1), after crosslinking (2), and after incubating for 48 h (3). In two-layer samples, 0% gelatin maintains approximately 25% normalized pore number after printing, after crosslinking, and after incubating. Increasing gelatin concentration to 1% *w*/*v* increased the normalized pore number to 50%, whereas 2% and 3% gelatin increased the normalized pore number to 98%. This increase is due to the increased viscosity that provides better printability. For the six-layer print (Figure 5d), 0% gelatin could not form a grid pattern, resulting in a normalized pore number of 0%. Comparing the six-layer with two-layer prints, the normalized pore number decreased by approximately 70% for 1% and 2% *w*/*v* gelatin due to filament collapse as a result of low bioink viscosity. However, no decrease was observed in the normalized pore number for 3% *w*/*v* gelatin after printing and after crosslinking, and only a 10 % decrease was recorded after incubation. Thus, 3% *w*/*v* gelatin in alginate yielded the best print quality for all three conditions.

The quantification results of pore area measurement with all bioinks for two-layer and six-layer prints are shown in Figure 5e and Figure 5f, respectively. These data show crosslinking does not highly affect the pore area. However, there is a slight increase in the pore area after incubation in all bio-ink types for both two- and six-layer designs. There was a 35% increase in the pore area of 3% *w*/*v* gelatin for the six-layer case. This increase in pore area can be a result of high temperature in the incubator which can expand the crosslinked solid pattern. The error bar was obtained from successfully printed pores and the average of three reputations of every single experiment.

### 3.5. Cell Viability Analysis

**Figure 6:**
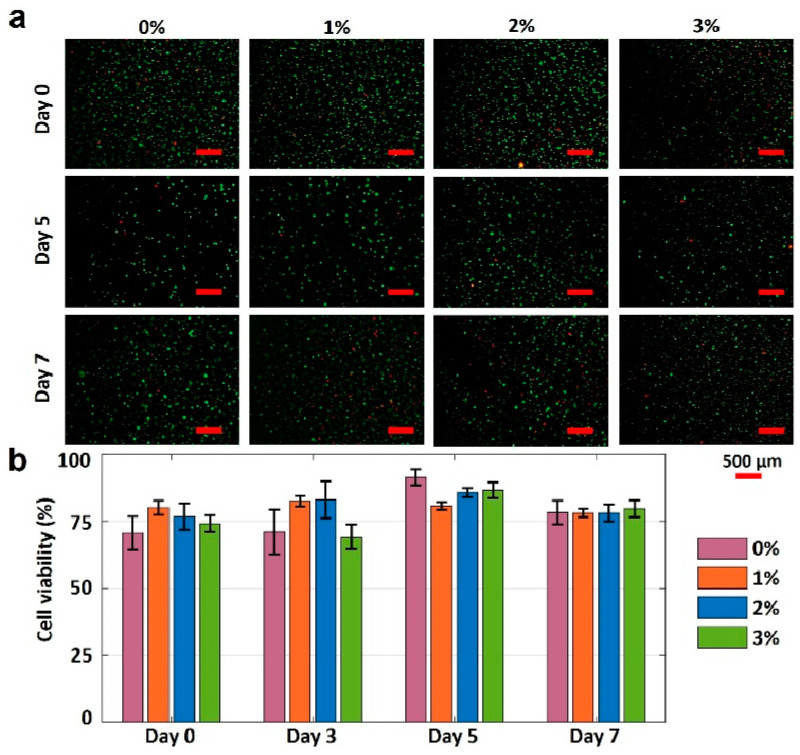
Characterization of cell viability. (**a**) Fluorescence images of National Institutes of Health (NIH) 3T3 mouse embryonic fibroblast cells in four different hydrogels show the cell viability after 0, 5, and 7 days. The green-stained (Calcein AM, 0.5 µL/mL) cells represent the alive cells. Red-stained (ethidium homodimer 1, 2 µL/mL) cells represent dead cells. (**b**) Quantification of the cell viability from live/dead image analysis. Error bars represent the standard deviation of three independent measurements. Scale bars are 500 μm. *p*-value is 0.03 (ANOVA: single-factor).

The gelatin concentration was varied to characterize the effects of gelatin concentration on the cell viability in 4% alginate. The fluorescence images of printed NIH 3T3 mouse embryonic fibroblast cells in four different bioinks over seven days are presented in Figure 6a. The cell viability results for varying gelation concentrations are depicted in Figure 6b. Pure alginate had the lowest cell viability of 70%. Adding gelatin increased the cell viability by approximately 15% on day 0. The cell viability of pure alginate was increased by 21% (to 91%) within five days of incubation, while the cell viabilities for other bioinks were increased by 1%, 11%, and 16% for the 1%, 2%, and 3% *w*/*v* gelatin concentrations, respectively (from day 0 and day 5). The lower increase in cell viability for bioinks with 1%, 2%, and 3% *w*/*v* gelatin (compared with pure alginate) can be attributed to their higher mechanical strength, which, in turn, can prohibit the nutrient and oxygen transfer to the cells. On day 7, the cell viability for all bioinks decreased, ranging from 4 to 14%, which can be attributed to the lack of nutrients and oxygen. In addition, it is observed that the highest cell viability belongs to the 3% gelatin on day 7. In conclusion, the proposed bioink, 3% *w*/*v* gelatin, is biocompatible and has the potential for 3D bioprinting of tissues and organs.

## 4. Conclusions

Developing multiple-component bioink formulations with high shape fidelity requires qualitative fidelity analysis and prediction methods. We have demonstrated two shape fidelity analysis techniques and a mathematical model using our custom bioprinter. The shape fidelity analysis methods developed herein offer readily accessible and replicable techniques to rapidly optimize new bioink formulations. They can help accelerate the development process by evaluating the shape fidelity performance of new bioinks. The first technique evaluates the shape fidelity based on the physical deformation of a single printed filament over a platform at two temperatures (room temperature (25 °C) and incubator temperature (37 °C)). The incubator can simulate the human body temperature to characterize the behavior of bioinks for possible in situ 3D bioprinting. It is shown that the deflection angle (θ1) decreased approximately three times faster at 37 °C (~5 min) compared with 25 °C (~15 min). Moreover, the slope of the 3D-printed filaments in an incubator decreased from ~0.028 to ~0.022 by increasing the alginate concentration from 2% to 8%, respectively, indicating higher shape fidelity for higher concentrations of bioink. The results from this technique provide an idea regarding how the bioink will behave in different temperatures.

The second technique focuses on improving the printability and structural fidelity of a bioink. Evaluating bioink formulations is crucial for printing more complex architectural constructs. The crosslinking and incubating process is the most delicate portion because maintaining shape fidelity is vital to achieving the desired pattern. The experiments illustrated that high shape fidelity is dependent on the concentration of the added gelatin. According to the rheological and mechanical data presented in Figure 4, the addition of the gelatin into the mixture positively affected the stability of the 3D-bioprinted structures. While pure 4% alginate could barely retain its grid pattern after printing, adding gelatin can increase the pore area from ~0.5 mm^2^ (for pure alginate) to ~1.5 mm^2^ (for 3% *w*/*v* gelatin) after incubation of a two-layer grid pattern. Moreover, the presence of gelatin improved the stability of the printed grid pattern against deformation after a 2-day incubation in a cell culture medium (Figure 5). The obtained results in this study are in consensus with previous investigations [35]. According to Figure 3, a higher concentration of alginate resulted in a lower deflection angle (i.e., higher shape fidelity) in hanging structures. A similar study with poloxamer 407 and poly(ethylene glycol) bioink illustrated an analogous negative correlation between poloxamer concentration and intensity of deformation [35]. As for the diffusion experiment, a higher concentration of alginate resulted in more viscous bioink and lower diffusion rate (i.e., better pattern retention) (Figure 5), which confirms the reports of higher Young’s modulus (i.e., higher shape fidelity) for a higher concentration of bioinks [35,49].

Finally, we demonstrated a method to determine the biocompatibility of proposed bioinks for use in tissue engineering. We characterized the change in cell viability of NIH 3T3 mouse fibroblast cells over time. It is demonstrated that adding 3% *w*/*v* gelatin to 4% alginate increased the cell viability by 15% on day 0. After a 5-day incubation, the cell viability of 3% *w*/*v* gelatin bioink increased by 16% compared with a 21% increase in the cell viability of pure alginate, indicating that the addition of gelatin did not have a considerable adverse effect on cell viability while improving the shape fidelity. Considering the accessibility and repeatability, these proposed methods should prove highly suitable for bioink development and help accelerate the process. Future studies can further focus on automized monitoring and optimization of the 3D-printing process (e.g., using machine learning techniques) to be able to detect defects in real time and apply required adjustments in printing parameters [1,55,56,57,58]. Future studies can be performed encompassing not only rheological challenges associated with the mixtures of various alginate bioinks but also the effects of environmental stimuli (e.g., the evaporation of the material, dynamic surface tension, and Marangoni stresses due to variation in boundary conditions).

This study presented a quantitative and reproducible method for shape fidelity assessment of bioinks, their printability, and biocompatibility (i.e., cell viability). However, the moderate resolution of the custom-designed 3D printer limited the evaluation of shape fidelity in complex designs. Furthermore, in a few cases, the incubation of printed patterns led to the swelling or contraction of printed filaments, resulting in inconsistencies in pattern fidelity readouts. In order to diminish the effect of possible readout errors in results, all experiments were repeated three times (standard deviations are presented by error bars in figures).

## Data Availability

Not applicable.

## Supplementary Materials

The following supporting information can be downloaded at: https://www.mdpi.com/article/10.3390/jfb13040225/s1, Figure S1: Characterization of filament deformation in room and incubator temperatures; Figure S2: Characterization of print quality and pore area for multiple-layer grid pattern.
