# Supplementary material for: Shape Fidelity Evaluation of Alginate-Based Hydrogels through Extrusion-Based Bioprinting
Source: J Funct Biomater. 2022 Nov 7;13(4):225. doi: 10.3390/jfb13040225 (PMC9680455; doi:10.3390/jfb13040225)
Supplement: Supplementary file 1 [file jfb-13-00225-s001.zip › jfb-1971278-supplementary.pdf]

Shape Fidelity Evaluation of Alginate-Based Hydrogels through Extrusion-Based Bioprinting

Mikail Temirel [1,2], Sajjad Rahmani Dabbagh [3] and Savas Tasoglu [3,4,5,6,*]

1 Department of Biomedical Engineering, University of Connecticut, Storrs, CT 06269, USA
2 Mechanical Engineering Department, School of Engineering, Abdullah Gul University, Kayseri 38080, Turkey
3 Department of Mechanical Engineering, Koç University, Sariyer, Istanbul 34450, Turkey
4 Koç University Arçelik Research Center for Creative Industries (KUAR), Koç University, Istanbul 34450, Turkey
5 Koç University Translational Medicine Research Center (KUTTAM), Koç University, Istanbul 34450, Turkey
6 Boğaziçi Institute of Biomedical Engineering, Boğaziçi University, Istanbul 34684, Turkey
* Correspondence: stasoglu@ku.edu.tr

Supplementary Figures:

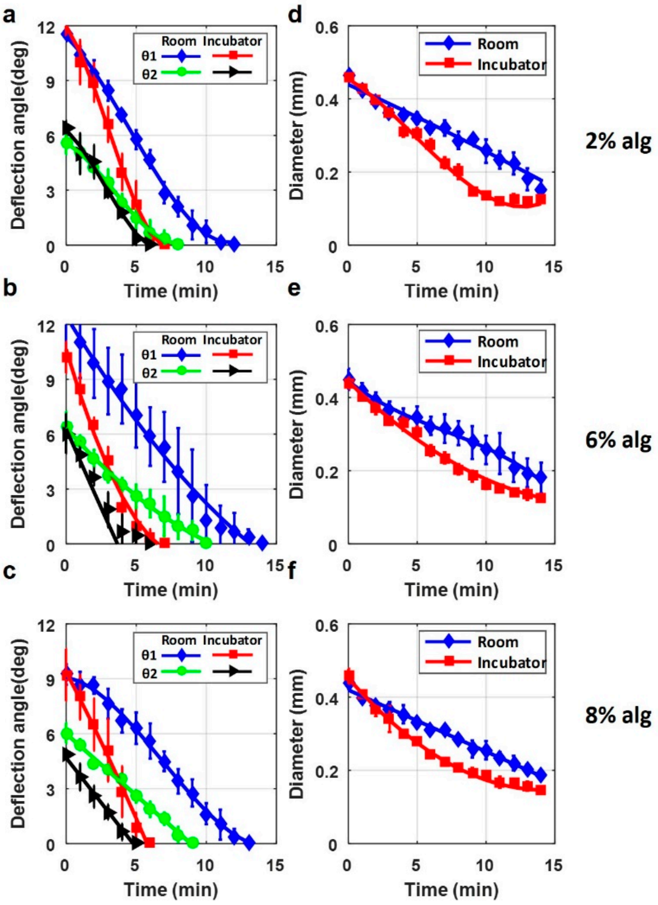

Figure S1. Characterization of filament deformation in room and incubator temperatures. (a-c) A comparison of two deflection angles on the filament in room (24°C) and incubator temperatures (37°C) over time (the experiment continued as long as filaments became straight) (a) for 2% alginate, (b) for 6% alginate, and (c) for 8% alginate (w/v) concentration. (d-f) A comparison on filament diameter changing in room and incubator temperature over time (d) for 2% alginate (e) for 6% alginate, and (f) for 8% alginate (w/v) concentration.

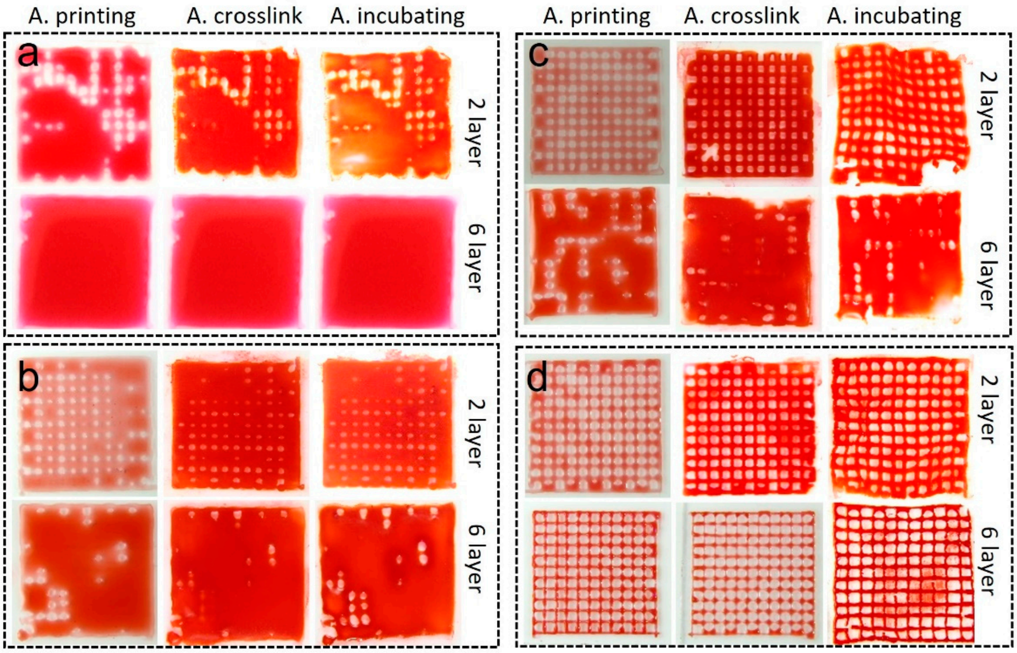

Figure S2. Characterization of print quality and pore area for multiple-layer grid pattern. (a) Images of 2-layer and 6-layer printed pattern for 4A0G bio-ink, (b) for 4A1G, (c) for 4A2G, and (d) for 4A3G. The first column is composed of the images that were taken immediately after printing, the second column represents 3D print outcomes after 2 mins of crosslinking in a calcium bath, and the third column depicts the same patterns after a 2-day incubation.
